# Molecular Characterization of *Cryptosporidium spp*. among Children in Rural Ghana

**DOI:** 10.1371/journal.pntd.0003551

**Published:** 2015-03-06

**Authors:** Daniel Eibach, Ralf Krumkamp, Hassan M. Al-Emran, Nimako Sarpong, Ralf Matthias Hagen, Yaw Adu-Sarkodie, Egbert Tannich, Jürgen May

**Affiliations:** 1 Bernhard Nocht Institute for Tropical Medicine (BNITM), Hamburg, Germany; 2 German Center for Infection Research (DZIF), partner site Hamburg-Borstel-Lübeck, Germany; 3 Kumasi Centre for Collaborative Research in Tropical Medicine (KCCR), Kumasi, Ghana; 4 Department of Tropical Medicine at the BNITM, German Armed Forces Hospital of Hamburg, Hamburg, Germany; 5 Kwame Nkrumah University of Science and Technology (KNUST), Kumasi, Ghana; Institut Pasteur, FRANCE

## Abstract

**Background:**

The relevance of *Cryptosporidium* infections for the burden of childhood diarrhoea in endemic settings has been shown in recent years. This study describes *Cryptosporidium* subtypes among symptomatic and asymptomatic children in rural Ghana to analyse subtype-specific demographic, geographical, seasonal and clinical differences in order to inform appropriate control measures in endemic areas.

**Methodology/Principal Findings:**

Stool samples were collected from 2232 children below 14 years of age presenting with and without gastrointestinal symptoms at the Agogo Presbyterian Hospital in the rural Ashanti region of Ghana between May 2007 and September 2008. Samples were screened for *Cryptosporidium spp*. by PCR and isolates were classified into subtypes based on sequence differences in the *gp60* gene. Subtype specific frequencies for age, sex, location and season have been determined and associations with disease symptoms have been analysed within a case-control study.

*Cryptosporidium* infections were diagnosed in 116 of 2232 (5.2%) stool samples. Subtyping of 88 isolates revealed IIcA5G3 (n = 26, 29.6%), IbA13G3 (n = 17, 19.3%) and IaA21R3 (n = 12, 13.6%) as the three most frequent subtypes of the two species *C*. *hominis* and *C*. *parvum*, known to be transmitted anthroponotically. Infections peak at early rainy season with 67.9% and 50.0% of infections during the months April, May and June for 2007 and 2008 respectively. *C*. *hominis* infection was mainly associated with diarrhoea (odds ratio [OR] = 2.4; 95% confidence interval [CI]: 1.2–4.9) whereas *C*. *parvum* infection was associated with both diarrhoea (OR = 2.6; CI: 1.2–5.8) and vomiting (OR = 3.1; 95% CI: 1.5–6.1).

**Conclusions/Significance:**

Cryptosporidiosis is characterized by seasonal anthroponotic transmission of strains typically found in Sub-Saharan Africa. The infection mainly affects young infants, with vomiting and diarrhoea being one of the leading symptoms in *C*. *parvum* infection. Combining molecular typing and clinical data provides valuable information for physicians and is able to track sources of infections.

## Author Summary

*Cryptosporidium spp*. are a frequent cause of diarrhoea worldwide. While both animal-to-human and human-to-human transmission has been reported from industrialized countries, human-to-human transmission clearly prevails in Sub-Saharan Africa. However, data on the distribution of zoonotic and human subtypes is limited for rural African regions, where children are in very close contact to animals. We conducted a case-control study with 2232 stool samples from symptomatic and asymptomatic children living in the rural Ashanti region of Ghana. The combination of molecular typing results and clinical data helped to untangle transmission routes and to analyze the association of clinical symptoms with specific *Cryptosporidium* subtypes. Our study results demonstrate seasonal transmission with no clusters of specific subtypes. All subtypes detected have so far been only encountered from human specimens, strongly suggesting a predominantly human-human transmission among children living in the rural Ghana, despite close contact to livestock. Therefore, public health control programmes need to primarily focus on hygienic conditions among young infants below the age of two years. Of interest for practicing physicians, vomiting is a frequent symptom, especially in *C*. *parvum* infections.

## Introduction

The parasitic protozoa *Cryptosporidium spp*. attracts attention with large epidemics in industrialized countries while being undiagnosed and neglected in many developing countries [1,2]. Cryptosporidiosis is an opportunistic infection in immune-compromised patients, which may results in severe and life-threatening diarrhoea [3–5]. In immunocompetent hosts the disease is usually self-limited, however it has been shown to induce weight loss, growth stunting, sustained impact on child development and increased case fatality [2,6–8].

In recent years a number of studies revealed the high proportion of *Cryptosporidium spp*. on the burden of diarrhoeal disease in endemic settings [1,2,8–12]. The Global Enteric Multicenter Study (GEMS) calculated the second highest attributable fraction on diarrhoeal disease for *Cryptosporidium spp*. in children less than 12 months [8]. Only in the last decade, increasing species-specific PCR-based diagnosis and sequencing-based subtyping methods (e.g. sequencing of the *gp60* gene) untangled routes of transmission of *Cryptosporidium infections* [13–16]. While zoonotic and anthroponotic transmission in industrialized countries varies by regions, human-to-human transmission of *Cryptosporidium hominis* and anthroponotic subtypes of *Cryptosporidium parvum* seem to be the dominant infection route in sub-Sahara Africa [9,13,17–23] However most studies from Sub-Sahara Africa were not conducted in rural areas, but in tertiary care hospitals or in metropolitan areas, where zoonotic transmission might be low.

Previous molecular-typing showed species and subtype specific reservoirs, as well as differences, in terms of diarrhoea manifestation, growth faltering and seasonality [12,13,24–26]. Cryptosporidiosis has been recognised as an important cause for childhood diarrhoea in Ghana [5,10]. Nevertheless, no further molecular studies have been performed in Ghana or its neighbouring countries. Molecular typing data on *Cryptosporidium spp*. for West Africa is up to now limited to publications from Nigeria [18,27,28].

This study aims to describe *Cryptosporidium* subtypes among symptomatic and asymptomatic children in rural Ghana and to analyse subtype specific routes of transmission, demographic characteristics as well as clinical differences. The combination of molecular typing results and clinical data will provide valuable information on reservoirs, endemic strains and pathogenicity.

## Methods

### Study site and study population

This study was performed in the context of a case-control study on the aetiology of diarrhoea in rural Ghana. Study site was the children’s Outpatients Department (OPD) of the Agogo Presbyterian Hospital (APH), a district hospital with 250 beds in the Asante Akim North municipality. This municipality has an estimated population of 142,400 inhabitants, spread over an area of 1,160 square kilometres. The region has a tropical climate and is mainly covered by secondary rain forest and cultivated land [29].

Children below 14 years of age visiting the hospital’s OPD between May 2007 and September 2008 with complaints of gastrointestinal symptoms (diarrhoea and/or vomiting) were included into the study and served as study cases. Diarrhoea was defined as at least three loose stools within the last 24 hours. Vomiting had to occur within the last 24 hours prior to the hospital visit. During the same study period, children of the same age visiting the OPD without any gastrointestinal symptoms, defined as absence of diarrhoea, vomiting or acute malnourishment were recruited as study controls. Each study participant had to provide one stool sample on the day of the hospital visit.

All participants were informed about the study’s purpose and procedures. Written informed consent was obtained from the parents or the guardian on behalf of the study children prior to study enrolment. Non-participation had no effect on the medical treatment provided. The Committee on Human Research, Publications and Ethics, School of Medical Science, Kwame Nkrumah University of Science and Technology, Kumasi, Ghana, approved the study design and the informed consent procedure.

### *Cryptosporidium spp*. subtyping and phylogenetic analysis

Immediately after stool collection, the sample was frozen at −20°C. DNA was extracted from the frozen stool samples using the QIAamp DNA Stool-Kit (Qiagen, Hilden, Germany). Extracted DNA was transported to Germany and subsequently a real-time polymerase chain reaction (RT-PCR) test was applied to identify *C*. *hominis/C*. *parvum* DNA as previously described [30]. The PCR targets the 18S small subunit ribosomal RNA gene and amplifies a 139 bp DNA fragment. As controls for DNA extraction and/or PCR inhibition each sample was contaminated with traces of phocine Herpesvirus-1 (PhHV-1) prior to faecal DNA extraction. RT-PCR for PhHV-1 was performed as previously described [31]. All PCR-confirmed *Cryptosporidium spp*. DNA samples were subject to amplification of a 850-bp fragment of the *gp60* gene using a nested PCR with the outer primers AL3531 and AL3535 and inner primers AL3532 and AL3534 as described before [20,32]. Amplicons were sent for purification and bidirectional sequencing to Eurofins (Hamburg, Germany). Forward and reverse sequences were assembled using Seqscape Software v3.0 (Applied Biosystems, USA) and subsequently aligned using the ClustalW program running within the BioEdit software package, version 7.0.9.0. Classification on species level was conducted by an alignment to reference sequences retrieved from the NCBI GenBank. The assignment to subtypes was performed according to the number of trinucleotide repeats (TCA, TCG or TCT) coding for the amino acid serine as described elsewhere [17]. The subtype IIcA5G3 was subdivided according to differences in the 3’ region of the *gp60* gene [17]. To further support the subtype classification, a neighbour-joining tree was built using the Kimura two-parameter model in MEGA 5 [33]. The reliability of all phylogenetic groupings was determined through a bootstrap resampling analysis (1,000 replicates). Six reference sequences of *Cryptosporidium spp*. subtypes from the NCBI GenBank database were included in the alignment as controls.

All sequences were uploaded to NCBI GenBank (Accession numbers KM538975—KM539062).

### Statistical and epidemiological analyses

Categorical variables were described as frequencies with corresponding percentages. Continuous variables were described using their mean and SD or the median and interquartile range (IQR), respective their distribution.

To describe gastrointestinal symptoms in children infected with *C*. *hominis* or *C*. *parvum* case-control studies for both species were conducted (S1 Dataset). In these analyses exposed children were infected with either *C*. *hominis* or *C*. *parvum*, un-exposed children were free of *Cryptosporidium* spp infection. Study outcomes were presence or absence of diarrhoea or vomiting, respectively. Age-adjusted odds ratios (OR), along with the 95% confidence intervals (CI) were calculated using the Mantel-Haenszel method. Missing values were excluded from the analysis, thus the denominators for some comparisons differ

The discriminatory power (D) was calculated as the average probability that the typing system differentiates two unrelated strains [34]. A typing method, which categorizes all different strains to different subtypes, gives a value of 1.0, while a value of 0.0 results from different strains being grouped into one single subtype.

All data analyses were performed with Stata 12 (StataCorp LP, College Station, USA).

## Results

Between May 2007 and October 2008 stool samples from 2,322 children were collected. Median age was 33 months (IQR 14–74) and 1,239 (53.4%) were male. In 116 (5.0%) samples *Cryptosporidium spp*. were detected by PCR.

**Fig 1:**
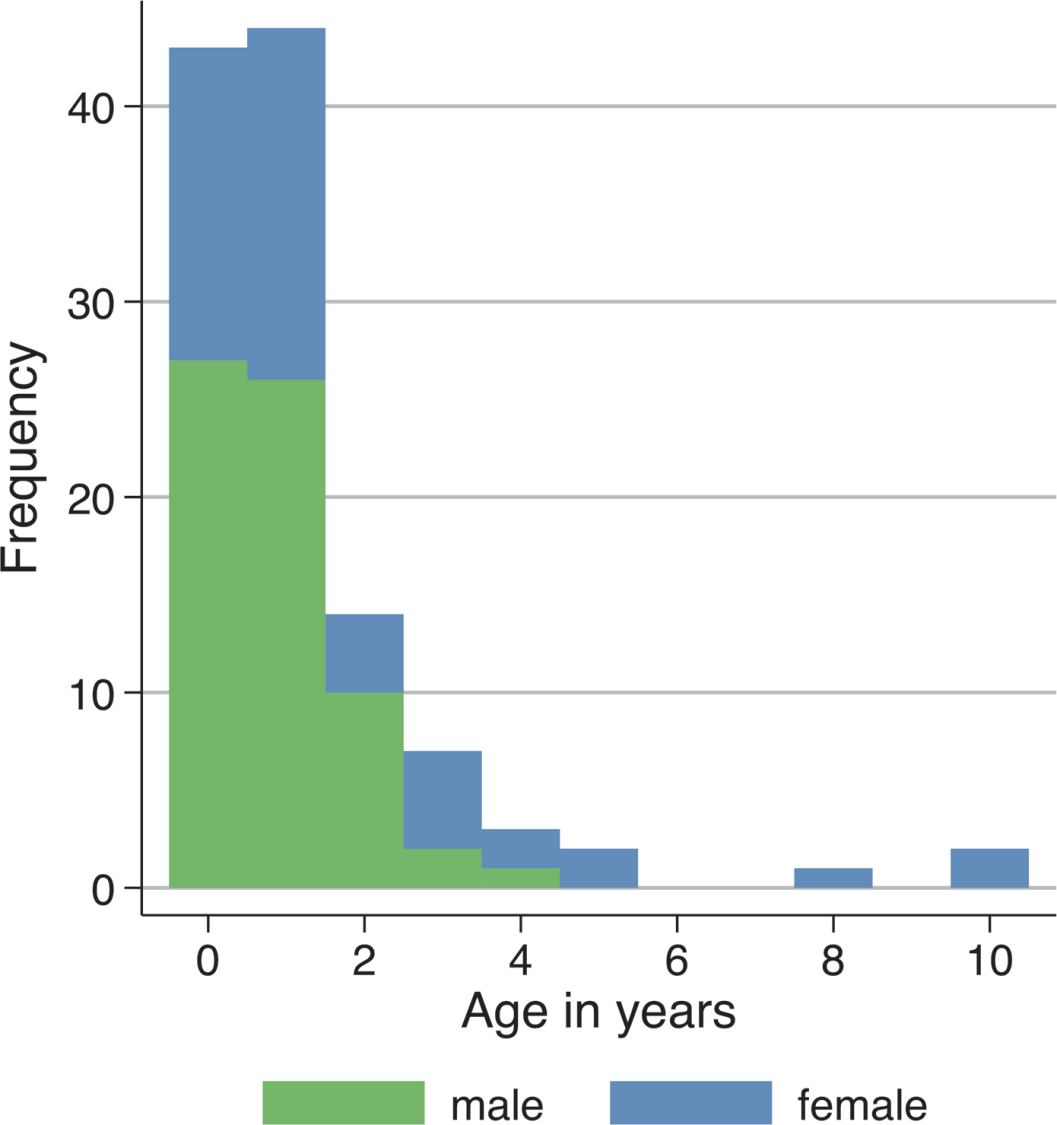
Number of *Cryptosporidium parvum/homini*s cases by age and sex (n = 116). The majority of patients are below the age of three (87.1%, n = 101) years with 56.9% (n = 66) of cases being male. The mean age for male subjects (0.85; SD 0.90) is lower than for females (1.74; SD 2.36).

**Fig 2:**
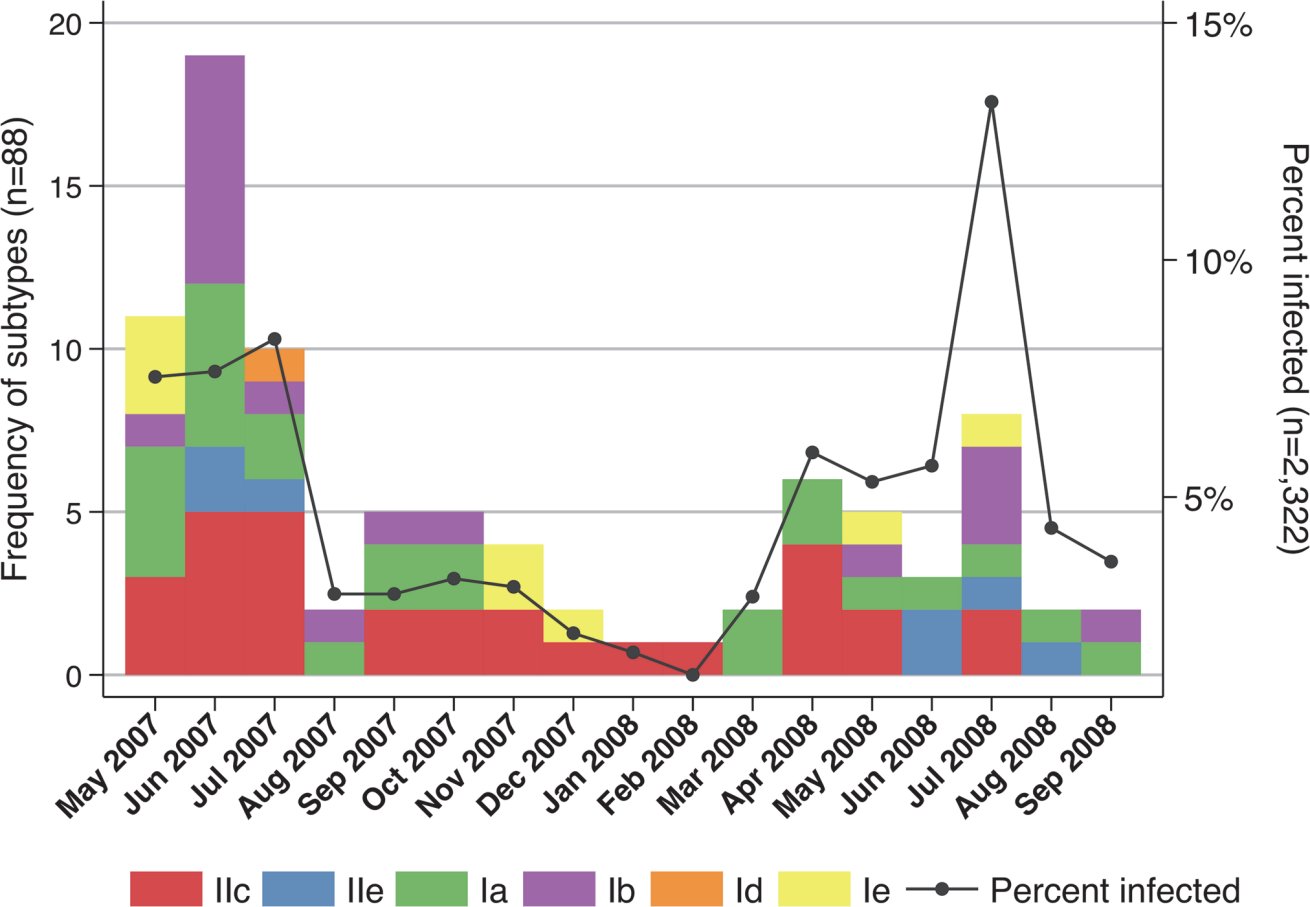
Distribution of Cryptosporidium subtypes (n = 88) and percentage of all study participants infected with Cryptosporidium spp. (n = 2,322) over the study period. The proportion of infected patients peak during rainy seasons from May to July 2007 and April to July 2008. No clusters of specific subtype families are observed over time.

Compared to the whole study group, the median age of *Cryptosporidium spp*. infected children was lower (median = 14 months; IQR 8–24). One hundred one (87.1%) and 87 (75.0%) patients were below the age of three and two years, respectively (Fig. 1). The majority of the cases were male (n = 66; 56.9%). The mean age for male subjects (0.85; SD 0.90) was lower than for females (1.74; SD 2.36). Patients were not equally distributed over the study period. In 2007, 10.5 patients/month were observed, while only 3.2 patients/month occurred in 2008. The proportion of infected patients peaked in the months May to July and April to July in 2007 and 2008 respectively, ranging between 5.3% (5/94) in May 2008 to 12.9% (8/62) in July 2008 (Fig. 2).

**Fig 3:**
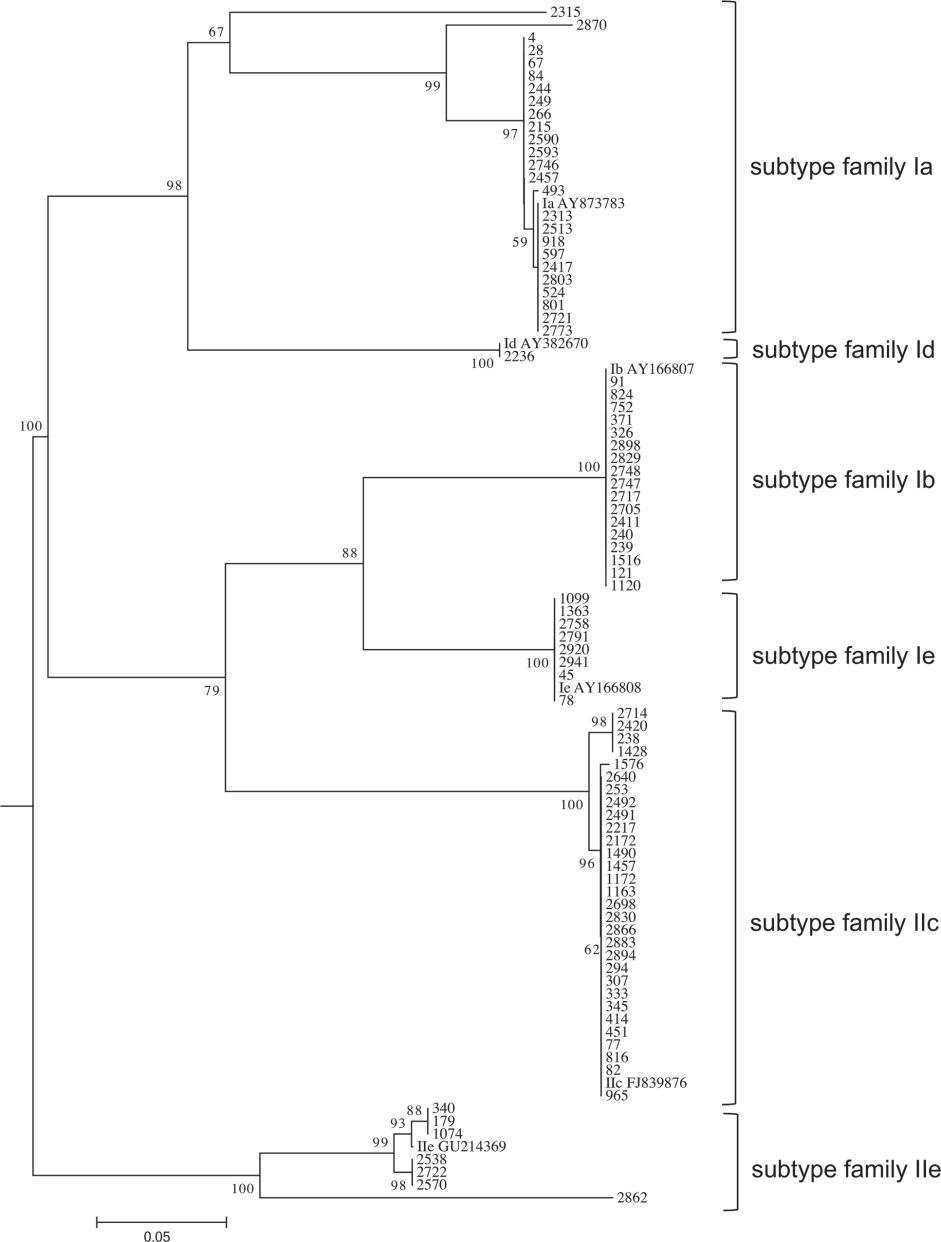
Phylogenetic analysis. Phylogenetic analysis of *C*. *hominis* and *C*. *parvum* subtypes and six reference strains with their respective accession numbers using neighbour-joining analysis of the gylcoprotein 60 (*gp60*) gene. Values on branches are percentage bootstrap values using 1,000 replicates. Only bootstrap values greater than 50% are shown.

For a subset of 88 (75.9%) out of the 116 *Cryptosporidium* patients/isolates the *gp60* gene was successfully amplified. Alignments to reference strains classified 51 (58.0%) strains as *C*. *hominis* and 37 (42.1%) as *C*. *parvum*. Four different *C*. *hominis* subtype families (Ia, Ib, Id, Ie) and two *C*. *parvum* subtype families (IIc, IIe) were identified. Further sub-classification led to 16 different subtypes of which the three most frequently observed types were IIcA5G3a (n = 26, 29.6%), IbA13G3 (n = 17, 19.3%) and IaA21R3 (n = 12, 13.6%) (Table 1). Ia was the genetically most diverse subtype family, consisting of eight different subtypes, while the other *C*. *hominis* subtype families comprise only one single subtype (IbA13G3, IdA15, IeAA11G3T3). The subtype families IIc and IIe contained two subtypes each (IIcA5G3a, IIcA5G3b and IIeA10G1, IIeA10G2, respectively). This heterogeneity resulted in a discriminatory power of 0.848 for subtyping of the *gp60* gene (Fig. 3).

The analysis of demographic patient data for the 88 sequenced strains showed that 23 (62.2%) of the *C*. *parvum* infected children were males, while the sex distribution for *C*. *hominis* infections was relatively equal (n = 27; 52.9%) (Table 1). Particularly, the *C*. *parvum* subtype family IIc (n = 20; 66.7% vs. n = 10; 33.3%) and the *C*. *hominis* subtype family Ia (n = 16; 64.0% vs. n = 9; 36.0%) dominated in male patients.

**Table 1:** Demographic characteristics of *Cryptosporidium parvum/hominis* subtypes (n = 88).

Subtype family	Subtypes	n (%)	mean age (sd)	male sex; n (%)
Ia	IaA15T1R3	1 (1.1)	1.0 (1.08)	16 (64.0)
	IaA15G1R4	1 (1.1)		
	IaA17	1 (1.1)		
	IaA18R3	4 (4.5)		
	IaA19R3	3 (3.4)		
	IaA21R3	12 (13.6)		
	IaA22R3	1 (1.1)		
	IaA24R3	2 (2.3)		
Ib	IbA13G3	17 (19.3)	1.0 (1.87)	6 (35.3)
Id	IdA15	1 (1.1)	0.0 (0.00)	1 (100.0)
Ie	IeA11G3T3	8 (9.1)	1.1 (1.73)	4 (50.0)
IIc	IIcA5G3a	26 (29.5)	1.0 (1.90)	20 (66.7)
	IIcA5G3b	4 (4.5)		
IIe	IIeA10G1	6 (6.8)	1.9 (1.95)	3 (42.9)
	IIeA10G2	1 (1.1)		

The median age was similar for *C*. *hominis* (13 months; IQR 8–18) and *C*. *parvum* (12 months; IQR 7–24). 90.2% (46/51) of *C*. *hominis* and 89.2% (33/37) of *C*. *parvum* infections occurred below the age of three. No age related differences were seen on subtype level.

The patients originated from 17 communities. 72.4% (63/87) of cases came from the three largest communities, which are Agogo (42 cases), Hwidiem (10 cases) and Konongo (11 cases). In the other 14 communities cases were observed sporadically. When looking at the distribution of subtypes over time for each community separately, not more than three cases of the same strain per month were detected.

No particular cluster of specific species or subtype families could be observed over time during or outside of the transmission peaks (Fig. 2)

Clinical information on the presence of diarrhoea and vomiting was available for 109 (94.0%) Cryptosporidiosis patients. Of those 73 (67.0%) and 50 (45.9%) patients had diarrhoea and vomiting, respectively. Both symptoms were reported from 41 (37.6%) patients. In the 2,206 (95%) children without *Cryptosporidium spp*. infection 929 (42.1%) and 785 (35.6%) had diarrhoea or vomiting, respectively. In this group 399 (18.1%) children showed both symptoms. This corresponds to 7.3% and 2.7% of *Cryptosporidium spp*. infections in children with and without diarrhoea symptoms and to 6.0% and 4.0% in children with and without vomiting, respectively.

The age-adjusted case-control study showed associations for *C*. *hominis* and diarrhoea (OR = 2.4; 95% CI: 1.2–4.9), whereas for *C*. *parvum* associations with vomiting (OR = 3.1; 95% CI: 1.5–6.1) and diarrhoea (OR = 2.6; 95% CI: 1.2–5.8) were shown (Table 2).

**Table 2:** *Cryptosporidium parvum/hominis* and their association with gastrointestinal symptoms.

**Clinical symptoms**	***Cryptosporidium* spp. negative (N = 2,206)**	***Cryptosporidium* spp. positive**			
		***C*. *hominis* (N = 45)**		***C*. *parvum* (N = 36)**	
	**n (%)**	**n (%)**	**OR (95%CI)**	**n (%)**	**OR (95%CI)**
**Diarrhoea^$^**	929 (42.1)	34 (75.6)	2.4 (1.2–4.9)	26 (72.2)	2.6 (1.2–5.8)
**Vomiting ^$^**	785 (35.6)	19 (42.2)	1.2 (0.7–2.3)	23 (63.9)	3.1 (1.5–6.1)

OR: Age-adjusted odds ratios, with 95% confidence intervals (CI), calculated using the Mantel Haenszel method

^$^for seven *Cryptosporidium spp*. positive children no clinical data was available.

## Discussion

All identified *C*. *parvum/hominis* subtypes from this study have not yet been identified in any animal samples, suggesting a dominating or even exclusive anthroponotic transmission in the rural Ashanti region of Ghana. The human-to-human transmitted *C*. *hominis* subtype families Ia, Ib, Id and Ie make up 58.0% of all *Cryptosporidium* strains found in this study. These four families were also the most commonly seen in children and HIV+ adults in other developing countries such as Peru, Malawi, Madagascar and India [12,24,26,35]. Within the family Ia this study identified eight different subtypes. This subtype diversity of family Ia is a common finding in developing countries [13]. The high heterogeneity shown in the phylogenetic tree, is thought to express intensive and stable anthroponotic cryptosporidium transmission in contrast to the more homogenous subtype distribution in animal samples or from countries with zoonotic transmission [13,20]. Interestingly, the very rare subtype IbA13G3 comprises 19.3% of all *Cryptosporidium* strains, being the second most frequent detected subtype in this study. So far this subtype has been only encountered in Peru, Nigeria and Cameroon and a west African origin has been assumed by some studies [24,27].

The zoonotically transmitted *C*. *parvum* subtype families IIa (with the worldwide prominently dominating subtype IIaA15G2R1), IId and IIl have not been found in the study population in Ghana. The two subtype families IIc and IIe, isolated in 37 (42.0%) patients, have been only detected in human specimens so far [13] and its anthroponotic route of transmission have been confirmed in the United States, Canada, Europe and Australia [13]. In developing countries most human *C*. *parvum* infections are due to subtype IIc. Additionally in African countries like Kenya, Malawi and Uganda the less frequent subtypes IIb and IIe have been detected [12,26,35–38], similar to the present study. The detected subtypes suggest that transmission in Ghana is exclusively human-to-human, however further studies in animals would be needed in this region to confirm this observation. Although in line with studies from other developing countries, the sole anthroponotic transmission is striking in the rural Ashanti region of Ghana, where children are in continuously close contact with farm animals that are most potentially infected as described from other areas [15].

The discriminatory power for *gp60* subtyping is high in the study setting (D = 0.848) compared to those from industrialized countries with less subtype diversity, making it a suitable tool for the detection of outbreaks in human-to-human transmission settings [16]. Nevertheless, no subtype specific clusters have been disclosed in any community during the study period.

Median age of *Cryptosporidium* infection was 14 months with males being infected at a younger age compared to females. Similar age distributions have been shown in many tropical countries. In a cohort study from Guinea 45% of all children experienced *Cryptosporidium* infections before the age of two years and a multicentre case-control study revealed the second highest attributable risk for moderate to severe diarrhoea for *Cryptosporidium spp*. among children below 12 months [8,39]. Prevalences are reported to be similar in urban and rural areas, but peak at an earlier age in rural households [40]. In concordance with this study, a higher infection risk for male children has been described in Nigeria and Guinea-Bissau [28,41]. This observation as well as the more frequent *C*. *parvum* infections in male children might be explained by host genetic factors or behavioural differences, however no conclusive evidence has been shown yet.

The study site normally experiences its main rainy season during the months April to July, which coincided with a peak of *Cryptosporidium* infections. Precipitation is known to be a strong seasonal driver for cryptosporidiosis in tropical countries, whereas in more temperate climates agricultural practices in spring (UK, New Zealand) or recreational water use during summer (USA, Canada) seem to be associated with infections [42,43].

Subtype specific associations with disease symptoms have been described before. In this study *C*. *parvum* reveals an association with diarrhoea and vomiting, while *C*. *hominis* shows to be primarily associated with diarrheal symptoms in comparison to children not infected with *Cryptosporidium* spp. Vomiting seems to be related to younger age groups with up to 70% of children affected as described in previous case control studies [7]. It has been postulated before, that *C*. *parvum* might be more virulent and infects a wider range of species [7]. In contrast, Cama et al. described the *C*. *hominis* subtype Ib as more virulent [12]. Due to low frequencies of each subtype no associations with disease symptoms could be assessed.

This study has some limitations. The HIV status of the study children could not be determined and therefore its influence on demography, subtype distribution and clinical symptoms remains unknown. However, the HIV prevalence in Ghana remains relatively low (1.4% in 2012; http://www.ghanaaids.gov.gh) and a previous study could not show a significant association between cryptosporidiosis and HIV in Ghana [44]. Subtyping of the *gp60* gene is not able to detect divergent *Cryptosporidium* species other than *C*. *parvum* and *C*. *hominis* (e.g. *C*. *meleagridis*, *C*. *canis*, *C*. *felis*). Therefore, *Cryptosporidium spp*. diversity in Ghana might even be higher than shown in this study. Further, co-infections with other enteric pathogens have not been analysed here, although previous studies have shown mechanistic interactions for enteric pathogens, such as *G*. *lamblia*/rotavirus co-infections [45].

### Conclusions

The absence of any subtype typically associated with zoonotic transmission of *C*. *parvum/hominis*, strongly suggests a predominantly anthroponotic transmission among children living in the rural Ashanti region of Ghana, despite close contact to livestock. Transmission is endemic with peaks in the rainy season, but without clonal clusters. As for other diarrhoeal diseases, public health control programmes need to primarily focus on hygienic conditions, particularly among young infants below the age of two years. Physicians should be aware that apart from diarrhoea, vomiting is a frequent symptom, especially in *C*. *parvum* infections. The finding of rare subtypes such as IbA13G3 and the IIe subtype family, previously mainly isolated from Sub-Saharan Africa, hints to the existence of regional endemic strains.

## Supporting Information

S1 Checklist STROBE checklist. (DOCX)

S1 Dataset Complete dataset for the case-control study. (ZIP)
