# Supplementary material for: Molecular Characterization of Cryptosporidium spp. among Children in Rural Ghana
Source: PLoS Negl Trop Dis. 2015 Mar 6;9(3):e0003551. doi: 10.1371/journal.pntd.0003551 (PMC4352007; doi:10.1371/journal.pntd.0003551)
Supplement: S1 Checklist — (DOCX) [file pntd.0003551.s001.docx]

STROBE Statement—checklist of items that should be included in reports of observational studies

The case-control study itself takes up only a small part of the manuscript. The main focus of this study is the molecular characterization of *Cryptosporidium* spp. in Ghana. Therefore items from this checklist might not appear as prominent in this manuscript as they would in exclusive case-control studies.

|  | | Item No. | | Recommendation | Line  No. | Relevant text from manuscript | |
| --- | --- | --- | --- | --- | --- | --- | --- |
| **Title and abstract** | | 1 | | (*a*) Indicate the study’s design with a commonly used term in the title or the abstract | 38 | …associations with disease symptoms have been analysed within a case-control study | |
|  |  |  |  | (*b*) Provide in the abstract an informative and balanced summary of what was done and what was found | 32  41 | See methodology  See principal findings | |
| Introduction | | | | | |  | |
| Background/rationale | | 2 | | Explain the scientific background and rationale for the investigation being reported | 105 | Cryptosporidiosis has been recognised as an important cause for childhood diarrhoea in Ghana. Nevertheless, no further molecular studies have been performed in Ghana or its neighbouring countries | |
| Objectives | | 3 | | State specific objectives, including any prespecified hypotheses | 128 | This study aims to describe *Cryptosporidium* subtypes among symptomatic and asymptomatic children in rural Ghana and to analyse subtype specific routes of transmission, demographic characteristics as well as clinical differences. | |
| Methods | | | | | |  | |
| Study design | | 4 | | Present key elements of study design early in the paper | 136 | This study was performed in the context of a case-control study on the aetiology of diarrhoea in rural Ghana…Children below 14 years of age visiting the hospital’s OPD between May 2007 and September 2008 with complaints of gastrointestinal symptoms (diarrhoea and/or vomiting) were included into the study and served as study cases | |
| Setting | | 5 | | Describe the setting, locations, and relevant dates, including periods of recruitment, exposure, follow-up, and data collection | 137 | Study site was the children’s Outpatients Department (OPD) of the Agogo Presbyterian Hospital (APH), a district hospital with 250 beds in the Asante Akim North municipality … Children below 14 years of age visiting the hospital’s OPD between May 2007 and September 2008… | |
| Participants | | 6 | | (*a*) *Cohort study*—Give the eligibility criteria, and the sources and methods of selection of participants. Describe methods of follow-up  *Case-control study*—Give the eligibility criteria, and the sources and methods of case ascertainment and control selection. Give the rationale for the choice of cases and controls  *Cross-sectional study*—Give the eligibility criteria, and the sources and methods of selection of participants | 1422 | Children below 14 years of age … with complaints of gastrointestinal symptoms (diarrhoea and/or vomiting) were included into the study 6nd served as study cases. Diarrhoea was defined as at least three loose stools within the last 24 hours. Vomiting had to occur within the last 24 hours prior to the hospital visit. During the same study period, children of the same age visiting the OPD without any gastrointestinal symptoms, defined as absence of diarrhoea, vomiting or acute malnourishment were recruited as study controls | |
|  |  |  |  | (*b*) *Cohort study*—For matched studies, give matching criteria and number of exposed and unexposed  *Case-control study*—For matched studies, give matching criteria and the number of controls per case | NA |  | |
| Variables | | 7 | | Clearly define all outcomes, exposures, predictors, potential confounders, and effect modifiers. Give diagnostic criteria, if applicable | 190 | In these analyses exposed children were infected with either *C. hominis* or *C. parvum*, un-exposed children were free of *Cryptosporidium* spp infection. Study outcomes were presence or absence of diarrhoea or vomiting, respectively. Age-adjusted odds ratios (OR), along with the 95% confidence intervals (CI) were calculated using the Mantel-Haenszel method. | |
| Data sources/ measurement | | 8* | | For each variable of interest, give sources of data and details of methods of assessment (measurement). Describe comparability of assessment methods if there is more than one group | 152 | See “Cryptosporidium spp. subtyping and phylogenetic analysis” | |
| Bias | | 9 | | Describe any efforts to address potential sources of bias | 199 | Adjusted regression used | |
| Study size | | 10 | | Explain how the study size was arrived at |  | This is a pathogen specific study in the context of a larger case-control study on diarrhea-association. Therefor no samples size calculation was done specifically for this project | |
| Quantitative variables | 11 | | Explain how quantitative variables were handled in the analyses. If applicable, describe which groupings were chosen and why | | 192 | Categorical variables were described as frequencies with corresponding percentages. Continuous variables were described using their mean and SD or the median and interquartile range (IQR), respective their distribution. | |
| Statistical methods | 12 | | (*a*) Describe all statistical methods, including those used to control for confounding | | 199 | Age-adjusted odds ratios (OR), along with the 95% confidence intervals (CI) were calculated using the Mantel-Haenszel method.  The discriminatory power (D) was calculated as the average probability that the typing system differentiates two unrelated strains | |
|  |  |  | (*b*) Describe any methods used to examine subgroups and interactions | | 199 | Age-adjusted odds ratios (OR), … were calculated using the Mantel-Haenszel method. | |
|  |  |  | (*c*) Explain how missing data were addressed | | 200 | Missing values were excluded from the analysis, thus the denominators for some comparisons differ. | |
|  |  |  | (*d*) *Cohort study*—If applicable, explain how loss to follow-up was addressed  *Case-control study*—If applicable, explain how matching of cases and controls was addressed  *Cross-sectional study*—If applicable, describe analytical methods taking account of sampling strategy | | NA |  | |
|  |  |  | (*e*) Describe any sensitivity analyses | | NA |  | |
| Results | | | | | | |  |
| Participants | 13* | | (a) Report numbers of individuals at each stage of study—eg numbers potentially eligible, examined for eligibility, confirmed eligible, included in the study, completing follow-up, and analysed | | 209, 222, 250 | Between May 2007 and October 2008 stool samples from 2,322 children were collected… For a subset of 88 (75.9%) out of the 116 *Cryptosporidium* patients/isolates the *gp60* gene was successfully amplified… Clinical information on the presence of diarrhoea and vomiting was available for 109 (94.0%) | |
|  |  |  | (b) Give reasons for non-participation at each stage | | 223, 250 | …was successfully amplified, …clinical information on the presence of diarrhoea and vomiting was available… | |
|  |  |  | (c) Consider use of a flow diagram | | NA |  | |
| Descriptive data | 14* | | (a) Give characteristics of study participants (eg demographic, clinical, social) and information on exposures and potential confounders | | 209 | First section of the result part + Table 1, figure 1 and 2 | |
|  |  |  | (b) Indicate number of participants with missing data for each variable of interest | | 521 | Table 2 | |
|  |  |  | (c) *Cohort study*—Summarise follow-up time (eg, average and total amount) | | NA |  | |
| Outcome data | 15* | | *Cohort study*—Report numbers of outcome events or summary measures over time | |  |  | |
|  |  |  | *Case-control study—*Report numbers in each exposure category, or summary measures of exposure | | 521 | Table 2 | |
|  |  |  | *Cross-sectional study—*Report numbers of outcome events or summary measures | | NA |  | |
| Main results | 16 | | (*a*) Give unadjusted estimates and, if applicable, confounder-adjusted estimates and their precision (eg, 95% confidence interval). Make clear which confounders were adjusted for and why they were included | | 260/521 | Table 2 and …The age-adjusted case-control study showed associations for *C. hominis* and diarrhoea (OR=2.5; 95% CI: 1.2-5.0), whereas for *C. parvum* associations with vomiting (OR=3.1; 95% CI: 1.5-6.1) and diarrhoea (OR=2.6; 95% CI: 1.2–5.8) were shown | |
|  |  |  | (*b*) Report category boundaries when continuous variables were categorized | | NA |  | |
|  |  |  | (*c*) If relevant, consider translating estimates of relative risk into absolute risk for a meaningful time period | | NA |  | |

| Other analyses | 17 | Report other analyses done—eg analyses of subgroups and interactions, and sensitivity analyses | 222-249 | The molecular characterization of Cryptosporidium spp. was presented in the first part of the result section. 215-242 |
| --- | --- | --- | --- | --- |
| Discussion | | | | |
| Key results | 18 | Summarise key results with reference to study objectives | 265 | All identified *C.* *parvum/hominis* subtypes from this study have not yet been identified in any animal samples, suggesting a dominating or even exclusive anthroponotic transmission in the rural Ashanti region of Ghana |
| Limitations | 19 | Discuss limitations of the study, taking into account sources of potential bias or imprecision. Discuss both direction and magnitude of any potential bias | 327 | This study has some limitations… |
| Interpretation | 20 | Give a cautious overall interpretation of results considering objectives, limitations, multiplicity of analyses, results from similar studies, and other relevant evidence | 267-326 | The molecular subtyping results and clinical symptoms are discussed with reference to the literature. |
| Generalisability | 21 | Discuss the generalisability (external validity) of the study results | 286 | The detected subtypes suggest that transmission in Ghana is exclusively human-to-human, however further studies in animals would be needed in this region to confirm this observation. Although in line with studies from other developing countries, the sole anthroponotic transmission is striking in the rural Ashanti region of Ghana, where children are in continuously close contact with farm animals that are most potentially infected as described from other area. |
| Other information | |  | | |
| Funding | 22 | Give the source of funding and the role of the funders for the present study and, if applicable, for the original study on which the present article is based |  | Funding statement submitted |

*Give information separately for cases and controls in case-control studies and, if applicable, for exposed and unexposed groups in cohort and cross-sectional studies.
